# Gewaltsamer Tod im Zusammenhang mit der COVID‑19‑Pandemie

**DOI:** 10.1007/s00194-022-00598-6

**Published:** 2022-11-11

**Authors:** S. Keimling, C. Babian, J. Dreßler

**Affiliations:** Institut für Rechtsmedizin, Universität Leipzig, Johannisallee 28, Haus H, 04103 Leipzig, Deutschland

**Keywords:** COVID-19, Pandemie, Übersterblichkeit, Suizid, Homizid, COVID-19, Pandemic, Excess mortality, Suicide, Homicide

## Abstract

**Hintergrund:**

Weltweit wird von einer durch die COVID-19-Pandemie bedingten Übersterblichkeit gesprochen. Ziel dieser Arbeit ist es zu prüfen, ob diese Übersterblichkeit nicht nur durch letale Krankheitsverläufe, sondern auch durch *pandemieassoziierte gewaltsame Todesfälle* verursacht wurde.

**Material und Methoden:**

In einer retrospektiven Studie wurden 825 Sektionsgutachten des Leipziger Instituts für Rechtsmedizin des Jahres 2020 ausgewertet: darunter 72 Suizide und 14 Homizide, welche auf einen Zusammenhang zur COVID-19-Pandemie untersucht wurden. Einbezogen wurden Ergebnisse der kriminalpolizeilichen Ermittlungen, sowie die Todesursachenstatistik der jeweiligen zuständigen Behörden der Jahre 2015–2020. Es erfolgte eine anonymisierte Dateneingabe. Die Daten wurden deskriptiv ausgewertet.

**Ergebnisse:**

Insgesamt waren 5 von 72 Suiziden (6,94 %) und einer von 14 Homiziden (7,14 %) durch die COVID-19-Pandemie motiviert. Die Anzahl der Suizide in Deutschland war in den Jahren 2015 bis 2020 insgesamt rückläufig; deutschlandweit war kein signifikanter Anstieg der Suizide 2020 erkennbar, wohingegen die Anzahl der Suizide in Sachsen im ersten Pandemiejahr 2020 um 8,7 % (nicht signifikant) stieg.

**Diskussion:**

In der untersuchten sächsischen Stichprobe waren ca. 7 % der Suizide und Homizide durch die COVID-19-Pandemie motiviert. Motive waren unter anderem: Ausgangsbeschränkungen, Reiseverbote, Angst vor einer Infektion mit dem COVID-19-Virus und pandemiebedingte Veränderungen im sozialen Umfeld.

Die „COVID-19-bedingte Übersterblichkeit“ ist damit auch auf pandemieassoziierte *gewaltsame* Todesfälle zurückzuführen.

Es wird beabsichtigt, die Untersuchungen für das zweite Pandemiejahr (2021) fortzuführen.

## Abstract (en)

**Background:**

Globally, there is talk of excess mortality due to the COVID-19 pandemic. The aim of this study is to examine whether this excess mortality is not only caused by fatal disease progression, but also by violent deaths associated with the pandemic.

**Material and methods:**

In a retrospective study, 825 autopsy reports from the Leipzig Institute for Forensic Medicine for the year 2020 were evaluated, including 72 suicides and 14 homicides, which were examined for a connection to the COVID-19 pandemic. The results of the criminal investigations and the statistics on the causes of death from the respective competent authorities for the years 2015–2020 were included.

Data were entered anonymously and evaluated descriptively.

**Results:**

Overall, 5 of 72 suicides (6.94%) and 1 of 14 homicides (7.14%) were motivated by the COVID-19 pandemic. The number of suicides in Germany fell overall between 2015 and 2020. Across Germany, there was no significant increase in suicides in 2020, whereas the number of suicides in Saxony rose by 8.7% (not significant) in the first year of the pandemic, 2020.

**Discussion:**

In the Saxony sample examined, around 7% of the suicides and homicides were motivated by the COVID-19 pandemic. Motives included travel and exit restrictions, fear of infection with COVID-19 and changes in the social environment caused by the pandemic.

The COVID-19-related excess mortality can therefore also be attributed to pandemic-associated violent deaths.

It is intent to continue research on data for the second year of the pandemic (2021).

## Einleitung

Seit Beginn der Pandemie im Jahr 2020 wurde durch COVID-19-Virusinfektionen in Deutschland im Vergleich zu dem präpandemischen Jahr 2019 eine durchschnittliche Übersterblichkeit von rund 5 % verzeichnet [1]. In Sachsen geht man im durchschnittlichen Vergleich mit den präpandemischen Jahren 2015–2019 von einer Übersterblichkeit von 13 % aus [2].

Diese Übersterblichkeit wird überwiegend auf infektionsbedingte letale Krankheitsverläufe zurückgeführt. Ziel dieser Arbeit ist es, anhand von Obduktionsberichten des Leipziger Institutes für Rechtsmedizin zu prüfen, ob diese Übersterblichkeit auch durch *pandemieassoziierte gewaltsame* Todesfälle verursacht wurde. In dieser Arbeit werden Suizide und Homizide als gewaltsame Todesfälle betrachtet.

## Material und Methoden

### Angaben aus amtlichen Statistiken

Als Datenbasis wurden die Todesursachenstatistik des Statistischen Bundesamtes und des Statistischen Landesamtes des Freistaates Sachsen der Jahre 2015–2019 („präpandemischer Zeitraum“) und des Jahres 2020 („pandemischer Zeitraum“) ausgewertet. Die Todesursachenstatistik für das Jahr 2021 lag noch nicht vor.

Erfasst wurden die Suizidzahlen je Monat und Jahr. Datenbasis für die Homizide war die Kriminalstatistik des Bundeskriminalamts.

### Einschluss- und Ausschlusskriterien

Die Sektionsgutachten des Leipziger Instituts für Rechtsmedizin des Jahres 2020 wurden anonymisiert ausgewertet. Das Versorgungsgebiet umfasst die Zuständigkeitsbereiche der Staatsanwaltschaften Leipzig, Chemnitz und Zwickau mit insgesamt 2.462.104 Einwohnern; das entspricht 60,8 % der Bevölkerung Sachsens (4.046.855 Einwohner).

In die Studie wurden alle Gutachten zu „nichtnatürlichen Todesfällen“ durch „Selbsttötung“ oder „Tod durch fremde Hand“ einbezogen.

Nicht einbezogen wurden Sektionsfälle mit „unklarer Todesursache“ oder „natürlichem Tod“, „Unfälle“, „Komplikationen nach medizinischer Behandlung“, sowie „Ereignisse, deren nähere Umstände unbestimmt“ waren. Die Daten wurden ohne Altersbeschränkungen erhoben.

### Datenerfassung zu studienrelevanten Sektionsfällen

Erfasst wurden Alter, Geschlecht, Sterbeort, Todesursache, Komorbiditäten, Motive, Art der Gewaltanwendung und Täter-Opfer-Beziehungen bei Homiziden. Berücksichtigt wurden dabei auch die Ergebnisse der polizeilichen Ermittlungen (z. B. Abschiedsbriefe, Zeugenaussagen, Ereignisortfeststellungen).

Die anonymisierte Erhebung und Verarbeitung dieser Daten wurde von der Ethikkommission genehmigt (015/22-ek).

Die Daten wurden klassifiziert und deskriptiv statistisch (Microsoft Excel, SPSS) ausgewertet. Einzelne Fälle werden kasuistisch präsentiert.

## Ergebnisse

### Amtliche Todesursachenstatistiken

**Figure 1:**
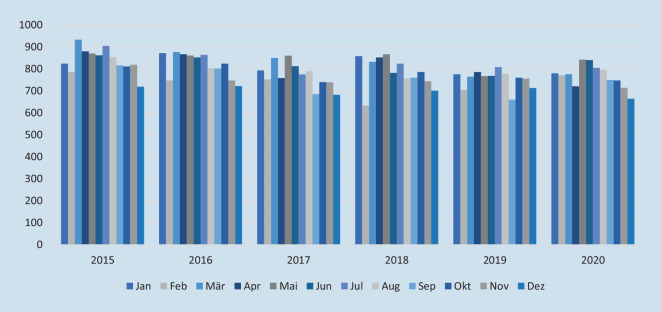

Die Anzahl der Suizide in Deutschland ist in den präpandemischen Jahren 2015–2019 fast durchgängig rückläufig, nur im Jahr 2018 wurden mehr Suizide als in den Vorjahren verzeichnet (Abb. 1).

Mithilfe eines Welch-*t*-Tests und zweier linearer Modelle zur Auswertung der präpandemischen Jahre 2015–2019 und des Pandemiejahres 2020 wurde unter Berücksichtigung des langfristigen Trends der letzten Jahre deutschlandweit kein signifikanter Anstieg oder Abfall der Suizidzahlen verzeichnet. Das erste Modell beruht auf einer einfachen linearen Regression. Die unabhängige Variable ist eine binäre Variable, die ausdrückt, ob der Datenpunkt vor oder nach der Pandemie liegt. Die abhängige Variable ist die Anzahl der Suizide. Das zweite Modell ist eine multiple lineare Regression. Zusätzlich zum ersten Modell enthält es noch eine weitere unabhängige Variable – in diesem Fall das Jahr.

**Figure 2:**
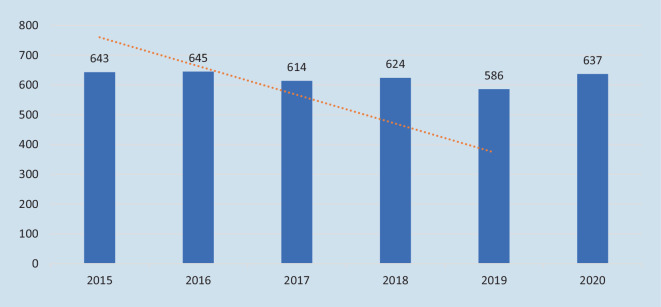

In Sachsen ist ebenfalls ein Abwärtstrend der Suizidzahlen in den Jahren 2015–2019 erkennbar – aber im Jahr 2020 stieg die Anzahl der Suizide in Sachsen um ca. 8,7 % zum Vorjahreswert. Der Anstieg war statistisch nicht signifikant (Abb. 2).

### Sektionen im Institut für Rechtsmedizin Leipzig

**Figure 3:**
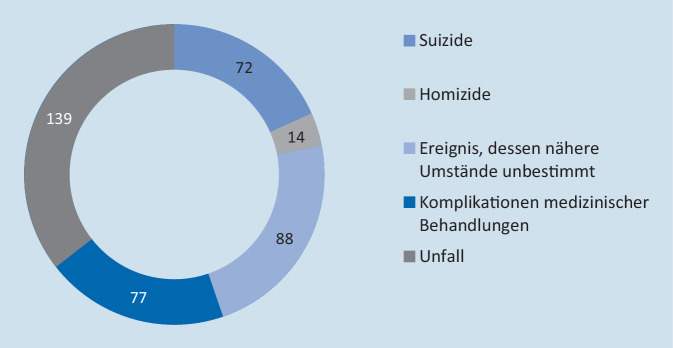

Am Leipziger Institut für Rechtsmedizin wurden im Jahr 2020 insgesamt 825 Sektionen durchgeführt. Dabei ergab die Obduktion in 390 Fällen eine nichtnatürliche Todesursache (Abb. 3).

Im Jahr 2020 waren bei 72 Fällen von Selbsttötungen die häufigsten Todesursachen: Erhängen (26,39 %), Vergiftungen (18,05 %), Bahnüberfahrungen (15,28 %) und Stürze in die Tiefe (12,5 %). Es überwog der Anteil der männlichen Suizidenten (74 %).

**Figure 4:**
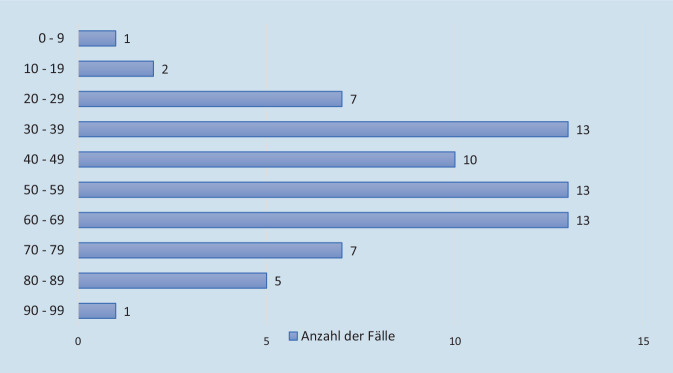

Die Altersverteilung der Suizident/-innen (männlich und weiblich) zeigt Abb. 4. Das durchschnittliche Sterbealter lag bei 51,3 Jahren, der Median bei 55 Jahren. Die meisten Suizide wurden im Alter zwischen 30 und 69 Jahren begangen. Die Spannweite beträgt 82 Jahre mit einem Minimum von 8 und einem Maximum von 90 Jahren.

### Komorbiditäten aller Suizide und derer mit Bezug zur COVID-19-Pandemie

Unter den 72 Suizident/-innen dieser Studie lag der Mittelwert bei 1,4 Vorerkrankungen (Komorbidität) pro Patienten, bei einer Spannweite von 0 bis 4 Vorerkrankungen. Am häufigsten lagen psychische und Verhaltensstörungen (56,9 %) vor, gefolgt von Herz-Kreislauf-Erkrankungen (23,6 %), Erkrankungen des Atmungssystems (12,5 %) und Erkrankungen des Verdauungssystems (11,1 %). Die Komorbiditäten werden in Tab. 1 dargestellt.

**Table 1:** 

Komorbidität nach ICD-10-Klassifikation	Aller gesicherten Suizide *n* = 72	Gesicherte Suizide mit Pandemiebezug *n* = 5
A00-B99 Bestimmte parasitäre und infektiöse Krankheiten	0 (0 %)	0 (0 %)
C00-D48 Neubildungen	5 (6,94 %)	1 (20 %)
D50-D90 Krankheiten des Blutes, der blutbildenden Organe	1 (1,39 %)	0 (0 %)
E00-E90 Endokrine, Ernährungs- und Stoffwechselkrankheiten	6 (8,33 %)	2 (40 %)
F00-F99 Psychische und Verhaltensstörungen	41 (56,94 %)	3 (60 %)
G00-H95 Krankheiten des Nervensystems und der Sinnesorgane	3 (4,17 %)	1 (20 %)
I00-I99 Krankheiten des Kreislaufsystems	17 (23,61 %)	3 (60 %)
J00-J99 Krankheiten des Atmungssystems	9 (12,5 %)	1 (20 %)
K00-K93 Krankheiten des Verdauungssystems	8 (11,11 %)	1 (20 %)
M00-M99 Krankheiten des Muskel-Skelett-Systems und des Bindegewebes	2 (2,78 %)	1 (20 %)
N00-N99 Krankheiten des Urogenitalsystems	4 (5,56 %)	2 (40 %)
R00-R99 Symptome und abnorme klinische Laborbefunde, die anderenorts nicht klassifiziert sind	3 (4,17 %)	0 (0 %)

Bei 5 von 72 Suizident/-innen bestand ein Bezug zur COVID-19-Pandemie; das entspricht 6,94 %. Unter 14 Homiziden bestand in einem Fall eine Assoziation zur COVID-19-Pandemie (7,14 %).

Unter den 5 Suizident/-innen mit Bezug zur COVID-19-Pandemie sind 3 an einer Intoxikation verstorben, ein/-e Patient/-in durch Strangulation und ein/-e Patient/-in verursachte den Tod mittels scharfer Gewalt.

#### Fallbeispiele für gewaltsame Todesfälle mit Bezug zur COVID-19-Pandemie

##### FALL 1 – 87-jähriger Mann mit Tod durch Digitoxinintoxikation.

Aus den Ermittlungsunterlagen der Polizei ging hervor, dass sich der 87-Jährige mit seinem Sohn gestritten habe. Dieser wollte, dass sein Vater das Haus aufgrund der COVID-19-Pandemie nicht mehr verlasse. Der Sohn schätzte die Pandemielage als zu gefährlich ein. Der Vater befand sich in psychiatrischer Behandlung, litt an Bluthochdruck und einer bluthochdruckbedingten Nierenerkrankung. Im Krankenhaus kündigte der Patient seinen Suizid an.

##### FALL 2 – 73-jähriger Mann mit Tod durch Strangulation.

Aus Familienbefragungen und Krankenakten ging hervor, dass der Patient an starken Schmerzen im Bereich des Karpaltunnels gelitten habe. Daraufhin sollte er im Krankenhaus operiert werden, allerdings wurde dieser Termin vom Patienten abgesagt, da seine Angst vor einer Infektion mit dem COVID-19-Virus im Krankenhaus zu groß war. Die Schmerzen belasteten seinen Alltag nach Angaben der Familie sehr, zuletzt sprach er im Zusammenhang mit seiner aktuellen Lebenssituation häufiger über Suizid.

##### FALL 3 – 54-jähriger Mann mit Tod durch Anwendung scharfer Gewalt in Form von Messerstichverletzungen im Halsbereich.

Der 54-Jährige litt unter Depressionen, Z. n. mehrfachen Suizidversuchen, einer Querschnittslähmung und einer Entzündung im Genitalbereich. Aus den polizeilichen Ermittlungen ging hervor, dass er sich durch die COVID-19-Pandemie in einer schlechten Lebenslage befand. Er gab gegenüber seiner Familie an, dass ihm die Trennung von seiner Ehefrau, die gesundheitlichen Probleme und die COVID-19-Pandemie zu schaffen machten. Der Patient fand nach Angaben durch die Pandemie keinen Arbeitsplatz und hatte 10 kg Körpergewicht verloren.

##### FALL 4 – 83-jähriger Mann mit Tod durch dekompensierter bluthochdruckbedingter Herzerkrankung nach Hydrochlorothiazidvergiftung.

Bei der Obduktion wurden oberflächliche Schnittverletzungen am linken Handgelenk festgestellt. Der Patient litt unter Diabetes mellitus, Bluthochdruck, einer Durchfallerkrankung, einem Magenkarzinom. Außerdem bestand der Verdacht auf ein Prostatakarzinom. Aus Familienbefragungen ging hervor, dass die Pandemie und die damit verbundenen Einschränkungen den Lebensalltag des Patienten stark belasteten.

##### FALL 5 – 38-jähriger Mann mit Tod durch Unterzuckerung nach Insulin- und Schmerzmittelintoxikation.

Anamnestischer Diabetes mellitus, Depressionen, Alkohol- und Nikotinabusus, Bluthochdruck, COPD und Z. n. zurückliegendem Suizidversuch. In diesem Fall lagen zwei Abschiedsbriefe vor. Der Suizident gab an, dass er durch die Trennung von seiner Lebensgefährtin keinen Lebenswillen mehr fand. Nach der Polizeibefragung der Lebensgefährtin konnte der Bezug zur COVID-19-Pandemie hergestellt werden. Diese berichtete über ihre Arbeit in einer Pflegeeinrichtung und das Tragen von COVID-19-Schutzmasken. Darüber habe sich der Lebensgefährte massiv aufgeregt und sei immer mehr in Verschwörungstheorien versunken, sodass die Trennung erfolgte.

##### FALL 6 – Homizid an einer 33-jährigen Frau durch Anwendung scharfer Gewalt in Form von Messerstichen gegen den Hals, Rumpf und die obere Extremität.

Die Frau verstarb an einem Hämatopneumothorax bei Verletzung der Brustschlagader und der Lungen nach mehrfachen Stichverletzungen des Brustkorbs und der Arme.

Ein Bezug zur COVID-19-Pandemie konnte hergestellt werden. Ursache des Homizids war ein eskalierter Streit, in dem es darum ging, dass der Frau das Leben in Deutschland nichts wert gewesen sei und sie zu ihrer Familie nach Afghanistan zurückreisen wollte. Die Lebensumstände unter Pandemiebedingungen stellten sie nicht zufrieden. Durch die COVID-19-Beschränkungen war eine Reise von Deutschland nach Afghanistan zu der Zeit nicht möglich, was immer wieder zu Auseinandersetzungen führte.

## Diskussion

Ob die COVID-19-Pandemie eine Auswirkung auf die Anzahl der Suizide hatte, wurde bisher vereinzelt, jedoch nicht für den Raum Sachsen, untersucht [3, 4]. Eine Assoziation der Pandemie explizit zu Homiziden wurde bisher noch nicht beschrieben.

Untersuchungen zu deren statistischer Abhängigkeit werden erschwert durch:

1. unklare Definition/Erfassung der COVID-19-Inzidenz [5],
2. geringe Anzahl statistisch erfasster Suizide/Homizide sowie unterschätzte Suizidraten durch unterschiedliche Erfassung in den Gesundheitsämtern [6],
3. übersehene Suizide/Homizide (keine ausreichende rechtsmedizinische Qualifikation der Ärzte bei Leichenschauen, Dunkelziffer ca. 1000 nichterkannte Homizide und 13.000 nichterkannte andere nichtnatürliche Todesfälle pro Jahr [7]),
4. geringe und regional unterschiedliche Sektionsquote von polizeilich bekannt gewordenen Suiziden/allgemeiner Rückgang der Obduktionszahlen [8].

Von 72 Suiziden, die im Jahr 2020 im Institut für Rechtsmedizin in Leipzig untersucht wurden, hatten 5 Fälle einen nachvollziehbaren Bezug zur COVID-19-Pandemie (6,94 %). Unter den 14 Homiziden gab es einen Fall mit Bezug zur COVID-19-Pandemie (7,14 %).

Der Pandemiebezug ergab sich aus diesbezüglichen Angaben in z. B. Abschiedsbriefen, Polizeiberichten oder aus Zeugenangaben.

In mehreren Fällen lagen Äußerungen zum kürzlich verlorenen Arbeitsplatz, einer momentan sehr schlechten Lebenslage (ökonomisch/psychisch) und existenziellen Ängsten vor. Diese Fälle wurden aus der Studie ausgeschlossen, da eine multifaktorielle Motivlage erkennbar war.

Es gab einen leichten, aber *nicht* signifikanten Anstieg der Suizidzahlen im Jahr 2020 für Deutschland und Sachsen (im Vergleich zu 2019).

Allerdings ist es möglich, dass dabei gegensätzliche Effekte eine scheinbare Konstanz der Suizidzahlen vortäuschen: In Japan seien die Suizidzahlen in den ersten 5 Monaten der Pandemie um 14 % gesunken, wohingegen die Suizidzahlen nach der zweiten Welle deutlich stiegen [4, 9]. Auch in anderen Studien wurde eine Stagnation oder sogar ein Abfall der Suizidzahlen in der frühen Pandemiephase festgestellt. Aus früheren Pandemien ist bekannt, dass es am Anfang einer Epidemie/Pandemie [10] oder Naturkatastrophe [11, 12] zu einem Rückgang der Selbsttötungen kommen kann – „Flitterwochenperiode“ oder „Honeymoon-Effect“ [13], weil der gesellschaftliche Zusammenhalt, der Optimismus, die Energie und der Altruismus in dieser Phase zunächst zunehmen und damit zunächst suizidpräventiv wirken können.

Andere Studien belegen die Zunahme der Suizidrate im Verlauf von längerfristigen Krisen: Während der Spanischen Grippe 1918/1919 stiegen die Suizidzahlen in den USA an [14]. Auch während des SARS-Ausbruchs in China 2003 wurde von einer erhöhten Suizidalität der älteren Bevölkerung und Menschen mit schwachen sozioökonomischen Status gesprochen [10]. Ein weiteres Paper beschreibt eine erhöhte Suizidalität während des Ebola-Ausbruchs in Afrika 2014–2016 [15].

In der vorliegenden Studie konnten nur die amtlichen Statistiken des ersten Jahres der Pandemie (2020) ausgewertet werden – möglicherweise zeigen deshalb die kommenden Jahre einen anderen Trendverlauf der *pandemieassoziierten* Suizidzahlen in Deutschland.

In dieser Studie dominierten unter den Komorbiditäten derjenigen Suizident/-innen mit Bezug zur COVID-19-Pandemie überwiegend die psychischen und Verhaltensstörungen sowie Herz-Kreislauf-Erkrankungen mit jeweils 30 %. Das entspricht den prädisponierenden Faktoren für Suizide in der präpandemischen Zeit [16].

Typische *pandemiebedingte* Auslöser für Suizide dürften sein: Einsamkeit und Isolation durch verstärkte soziale Distanzierung, Reisebeschränkungen, Quarantänebestimmungen und ein mangelnder Zugang zu technischen Mitteln und damit ein insuffizienter Zugriff auf aktuelle Informationen zur Pandemie. Vorbestehende krankhafte Ängste und Unsicherheiten werden durch die zunächst unbekannten Hintergründe der Infektion, Fehlinformationen, widersprüchliche und reißerische Medienberichterstattungen verstärkt. Soziale Stigmata, Vorurteile, Beschuldigungen und Gemeinschaftsgefühle begünstigen das Ausgrenzen bestimmter Bevölkerungsgruppen wie z. B. von Infizierten oder Impfverweigerern. Diese Bevölkerungsgruppen können durch die Ausgrenzung eher in soziale Isolation fallen.

Pandemiebedingte Einschränkungen der ambulanten und stationären medizinischen Behandlung sowie der psychosozialen Hilfsangebote sind weitere Risikofaktoren für ein steigendes Suizidrisiko bei prädisponierten Patienten.

Auch ökonomische Folgen der Pandemie wie z. B. Arbeitsplatzverluste, Einkommenseinbußen, finanzielle Zukunftsängste fördern das Risiko für Gewaltdelikte [17, 18].

Weitere pandemietypische Besonderheiten sind die erhöhte Kontaktzeit zwischen Partnern und Kindern durch Lockdown, Homeoffice, Homeschooling und ein verändertes Konsumverhalten (Alkohol, Drogen) – es resultieren anhaltende Überforderungssituationen, die sowohl selbst- als fremdgefährdendes Verhalten begünstigen.

Es lassen sich vulnerable Gruppen identifizieren, die in Krisenzeiten besonders geschützt werden müssen. Das sind Heranwachsende, alte Menschen (erhöhtes Isolationsrisiko, Einsamkeit), Gesundheitspersonal an vorderster Front (z. B. Ärzte, Krankenschwestern, die Gesundheitsaufsicht, Lieferanten und Freiwillige), Migranten und Obdachlose, Menschen in Armut oder mit einem niedrigen sozioökonomischen Status [19].

Präventiv gegen Gewaltdelikte – insbesondere *pandemiebedingte* Gewalttaten – wirken:

- Entwicklung psychologischer Konzepte zum Umgang mit pandemiebezogenen Ängsten und psychischen Störungen [20, 21],
- Konzepte zum spielerischen Lernen von pandemiespezifischem Wissen für Kinder [20],
- einfache und verständliche Übermittlung der wichtigsten Informationen zum Pandemiegeschehen [21],
- Kontakte zu Angehörigen, psychologischen Mitarbeitern oder anderen Vertrauenspersonen aufrechtzuerhalten [21],
- Verweise bei Auffälligkeiten im Krankenhaus oder in der hausärztlichen Ambulanz auf spezielle Organisationen wie Frauenhäuser, Hilfetelefone etc.,
- individuelle Stressbewältigungskonzepte,
- Öffentlichkeitsarbeit zum Thema „häusliche Gewalt“.

## Limitationen

Die offiziellen Veröffentlichungen des Landesamts für Statistik Sachsen und des Bundeskriminalamts lagen für das Pandemiejahr 2021 noch nicht vor. Es wird beabsichtigt, die Studie für das Pandemiejahr 2021 fortzuführen.

Nicht alle Suizidenten hinterlassen einen Abschiedsbrief, wodurch genaue Beweggründe nicht zu erfassen sind. Aussagen in Abschiedsbriefen oder Angehörigenbefragungen waren häufig nicht eindeutig genug, um sie der Pandemie zuordnen zu können.

Polizeiliche Ermittlungsunterlagen werden jeweils nur mit dem aktuellen Stand zum Zeitpunkt der Obduktion übermittelt, die Ergebnisse von Nachermittlungen lagen später nicht zur Auswertung vor.

Begleiterkrankungen werden im Obduktionsschein zumeist mit „V. a.“ (Verdacht auf) oder „n. A.“ (nach Angabe) versehen, sodass dabei nicht immer von einer absoluten Korrektheit ausgegangen werden kann.

## Fazit

Erstmals wurde der Zusammenhang zwischen der COVID-19-Pandemie im Jahr 2020 und gewaltsamen Todesfällen (Suizide, Homizide) im Sektionsgut des Leipziger Instituts für Rechtsmedizin untersucht. Da keine repräsentative Stichprobe vorlag, wurden die Untersuchungsergebnisse im Kontext der amtlichen Todesursachenstatistiken für Deutschland und Sachsen diskutiert.

Im Untersuchungsgut waren 5 von 72 Suiziden (6,94 %) und einer von 14 Homiziden (7,14 %) durch die COVID-19-Pandemie motiviert.

Die Anzahl der Suizide in Deutschland war in den Jahren 2015–2020 insgesamt rückläufig; in Sachsen stieg die Anzahl der Suizide im ersten Pandemiejahr 2020 um 8,7 %.

Es konnte kein signifikanter Anstieg der Suizidzahlen in Deutschland im ersten Pandemiejahr (2020) aufgezeigt werden. In der untersuchten sächsischen Stichprobe waren ca. 7 % der Suizide und Homizide durch die COVID-19-Pandemie motiviert – die „coronabedingte Übersterblichkeit“ ist damit auch auf *pandemieassoziierte* gewaltsame Todesfälle zurückzuführen.
